# 

**Published:** 1882-12

**Authors:** 


### CONTENTS:

PAGE.

Original Communications:

The Uses of Ergot in Obstetrics. By Chas. G. Stockton, M. D., . . . . . 193
The Abuses of Ergot in Obstetrics. By C. C. Frederick, M. D., . . . . . 198

Clinical Reports:

A Clinical Contribution to Treatment of Urethral Stricture. By Frank Dudley Beane, A. M., M. D., . . . . . 207
Physiological Action of Spinal Accessory Demonstrated by Injury of its Filaments. Reported by W. S. Tremaine, M. D., U. S. A., . . . . . . . . 218
A New Treatment for Hemorrhoids—A Pile Pessary. Reported by Thomas Lothrop, M. D., . . . . . . . . . 219

PAGE.

Selections:

Sick Headache, . . . . . . . 222

Pulvis Doveri, . . . . . . . 223

Correspondence:

Editorial Correspondence. By A. R. D., . 224
Malaria in Skin Diseases—A Correction. By Lunsford P. Yandell, . . . . 229

Society Reports:

Buffalo Medical and Surgical Association, . 231

Editorial:

Our Public Institutions, . . . . . 232

Editorial Notes, . . . . . . . 234

Reviews: . . . . . . . . 237

